# The Good Talk! A Serious Game to Boost People’s Competence to Have Open Conversations About COVID-19: Protocol for a Randomized Controlled Trial

**DOI:** 10.2196/40753

**Published:** 2023-03-08

**Authors:** Javier A Elkin, Michelle McDowell, Brian Yau, Sandra Varaidzo Machiri, Shanthi Pal, Sylvie Briand, Derrick Muneene, Tim Nguyen, Tina D Purnat

**Affiliations:** 1 Digital Health and Innovation Department, World Health Organization Geneva Switzerland; 2 Harding Center for Risk Literacy Faculty of Health Sciences Brandenburg University of Potsdam Potsdam Germany; 3 Max Planck Institute for Human Development Berlin Germany; 4 Department of Pandemic and Epidemic Preparedness and Prevention World Health Organization Health Emergencies Programme World Health Organization Geneva Switzerland; 5 Vaccine Safety Project, African Field Epidemiology Network Kampala Uganda; 6 Department of Regulation and Prequalification, World Health Organization Geneva Switzerland

**Keywords:** vaccine hesitancy, communication, serious game, conversation skills, self-efficacy, behavioral intentions, COVID-19

## Abstract

**Background:**

Vaccine hesitancy is one of the many factors impeding efforts to control the COVID-19 pandemic. Exacerbated by the COVID-19 infodemic, misinformation has undermined public trust in vaccination, led to greater polarization, and resulted in a high social cost where close social relationships have experienced conflict or disagreements about the public health response.

**Objective:**

The purpose of this paper is to describe the theory behind the development of a digital behavioral science intervention—*The Good Talk!*—designed to target vaccine-hesitant individuals through their close contacts (eg, family, friends, and colleagues) and to describe the methodology of a research study to evaluate its efficacy.

**Methods:**

*The Good Talk!* uses an educational serious game approach to boost the skills and competences of vaccine advocates to have open conversations about COVID-19 with their close contacts who are vaccine hesitant. The game teaches vaccine advocates evidence-based open conversation skills to help them speak with individuals who have opposing points of view or who may ascribe to nonscientifically supported beliefs while retaining trust, identifying common ground, and fostering acceptance and respect of divergent views. The game is currently under development and will be available on the web, free to access for participants worldwide, and accompanied by a promotional campaign to recruit participants through social media channels. This protocol describes the methodology for a randomized controlled trial that will compare participants who play *The Good Talk!* game with a control group that plays the widely known noneducational game *Tetris*. The study will evaluate a participant’s open conversation skills, self-efficacy, and behavioral intentions to have an open conversation with a vaccine-hesitant individual both before and after game play.

**Results:**

Recruitment will commence in early 2023 and will cease once 450 participants complete the study (225 per group). The primary outcome is improvement in open conversation skills. Secondary outcomes are self-efficacy and behavioral intentions to have an open conversation with a vaccine-hesitant individual. Exploratory analyses will examine the effect of the game on implementation intentions as well as potential covariates or subgroup differences based on sociodemographic information or previous experiences with COVID-19 vaccination conversations.

**Conclusions:**

The outcome of the project is to promote more open conversations regarding COVID-19 vaccination. We hope that our approach will encourage more governments and public health experts to engage in their mission to reach their citizens directly with digital health solutions and to consider such interventions as an important tool in infodemic management.

**International Registered Report Identifier (IRRID):**

PRR1-10.2196/40753

## Introduction

### Background

Vaccine hesitancy is one of the many factors impeding public health efforts to control the COVID-19 pandemic [1,2]. High vaccine uptake, especially in vulnerable and high-risk groups, is crucial to reduce severe disease hospitalization and death; however, vaccination rates differ markedly across countries (eg, 50% in Russia, 77% in Germany, and 91% in Singapore [3-5]). The World Health Organization (WHO) defines vaccine hesitancy as “a motivational state of being conflicted about, or opposed to, getting vaccinated; this includes intentions and willingness” [6], and it is a dynamic state that can evolve over the course of a pandemic. Reasons for vaccine hesitancy include a misunderstanding regarding COVID-19, a general mistrust of vaccination, safety and effectiveness concerns, concerns about the rapid development and testing of the vaccine, lack of awareness of the social benefits of vaccination, and logistical or opportunity-related factors [2,7-11]. These concerns have been exacerbated by the COVID-19 *infodemic*, in which “too much information including false or misleading information in digital and physical environments during a disease outbreak,” can lead to confusion and loss of trust in health authorities and public health responses [12]. COVID-19 misinformation has not only undermined public trust in vaccination [13] but has also led to a high social cost where close social relationships have experienced conflict or disagreements about pandemic control efforts [14-16]. In response, the WHO and others have called for greater application of behavioral science interventions targeting behavioral change and to support individuals in dealing with the psychological burdens associated with pandemic prevention and response [17-19]. For instance, evidence-based interventions that can help strengthen the resilience of individuals and communities to misinformation and other factors affecting trust in information will be a valuable tool for infodemic management [20].

### Objectives

The aim of this paper is to describe the theory behind the development of a new digital behavioral science intervention—*The Good Talk!*—designed to target vaccine-hesitant individuals through their close contacts (eg, family, friends, and colleagues) and to describe the methodology of a research study to evaluate its efficacy. *The Good Talk!* uses a serious game—a game that does not have “entertainment, enjoyment or fun as their primary purpose” [21]—to boost the competences of vaccine advocates to have open conversations regarding COVID-19 with their close contacts who are vaccine hesitant. Serious games have been used as educational tools to build skills, including teaching perspective taking to help students improve their open conversation skills [22] and inoculating people against misinformation (eg, the *Bad News* game [23]). Peer-led initiatives have been used to address mistrust in other domains, and communicating through relatable and trusted sources can lead to more acceptable and personal information exchanges, promoting prosocial behaviors when common identities are shared [1,19]. Furthermore, by focusing on boosting open conversation skills rather than addressing a specific cause of vaccine hesitancy, the approach has the potential to address a variety of reasons why an individual is vaccine hesitant [1,24] as well as build transferrable skills that can be applied across a variety of situations [25]. *The Good Talk!* is publicly available worldwide and will be evaluated in a preregistered randomized trial. Evaluating the impact of the intervention will be the subject of a future publication.

### Vaccine Hesitancy, Polarization, and the Social Impacts of the COVID-19 Pandemic

In 2019, the WHO identified vaccine hesitancy as one of the 10 threats to public health [26]. Despite strong and sustained evidence of the efficacy of vaccinations, perceptions of the safety and effectiveness of some vaccines have varied over the past years [27,28], affecting vaccine uptake [29] and contributing to disease outbreaks [24]. The influence of vaccine hesitancy on public health efforts to manage disease outbreaks has been accentuated during the COVID-19 pandemic, in which vaccine uptake in some countries has been low or lagging despite high vaccine access [3,4,30,31]. Consequently, longer durations of public health and social measures to reduce COVID-19 transmission are required, resulting in a variety of economic, health, and social costs with substantial psychological burden on individuals and communities [19,32].

The social costs of vaccine hesitancy have also been fueled by the COVID-19 infodemic, in which an overabundance of information has made it more difficult for the public to differentiate between correct and misinformation. When the public’s questions and concerns are left unanswered, information voids occur, which can lead to widespread false and misleading information. For instance, searches involving antivaccination rhetoric increased during the COVID-19 pandemic as people sought information about vaccine safety [33]. The infodemic has diluted trust in institutions, leading to confusion and disagreement about vaccination and safety measures [34,35], and has resulted in an increasingly polarized environment in which people avoid engaging with those who hold opposing or divergent views, negatively affecting interpersonal relationships [36].

For instance, 56% of participants in a nationwide UK-based study reported having arguments, feeling angry, or falling out with others because of discussions surrounding COVID-19 [16]. In addition, people report relationship strain, interpersonal conflict, and increased isolation associated with sharing differing opinions or information about COVID-19 with family members or friends or avoiding conversations altogether to prevent conflict [14,15]. Thus, interventions to help people develop skills to discuss potentially polarizing topics and maintain supportive relationships with those who hold different opinions are needed to reduce the negative social costs of the pandemic [15]. Furthermore, considering the evolving nature of COVID-19—both in terms of the emergence of new variants and cyclical gravity because of seasons—governments have imposed, lifted, and changed regulations frequently. As such, the topic of COVID-19 and vaccinations will hold relevance for many years to come [37].

### Intervention Goal: Initiate One-on-One Open Conversations About COVID-19 Vaccination

We sought to develop an evidence-based behavioral science intervention that could address diverse drivers of vaccine hesitancy while also supporting individuals in managing the psychological burden of the COVID-19 pandemic. Vaccine hesitancy is context specific and falls along a dynamic continuum, with motivation toward vaccination existing on a spectrum of those who are fully, partially, or not yet vaccinated [6,38,39]. As such, one-size-fits-all public health communication strategies are unlikely to reach all such individuals and will require a tailored, targeted, and multifaceted approach [1,24].

Reviews of a broad range of intervention approaches for addressing vaccine hesitancy report a lack of evidence or mixed findings on the effectiveness of many interventions [6,40-42], with dialogue-based approaches being among the most effective strategies [40]. Dialogue-based approaches aim to facilitate and encourage open communication to understand a specific audience and their underlying concerns [40]. Open communication–based approaches have the potential to address the negative effects of the COVID-19 infodemic on polarization and interpersonal relationships. For instance, exposure to divergent views through interpersonal discussion can weaken the association between selective exposure—exposure to content and groups that reinforce one’s opinions—and polarization [43]. Open communication is important for showing respect and empathy toward a communication partner, which can reduce defensiveness, increase receptiveness, and pave the way for open-minded conversations in which speakers are willing to share their views, consider new information, and potentially change their attitudes [44].

In addition, personalized communication can increase COVID-19 vaccine uptake, and these interventions are most effective when tailored to and delivered by a trusted peer or community member [6,42,45]. Advocating normative or prosocial behavior is more effective when a member of one’s social network models the behavior and there is an expectation of social approval [19]. Social approval is important, as people have a strong need to belong [46] and people listen to others in their social environment [47]. Accordingly, vaccine-hesitant individuals may be more likely to listen to and trust their close ties, as public trust in health institutions among vaccine-hesitant individuals is exceedingly low [19,48], and peer-led initiatives have shown positive outcomes when addressing mistrust in other domains [1]. Peer-led initiatives capitalize on social networks to communicate and can influence social norms to promote behavior change [41]. Furthermore, communicating through trusted and relatable sources can facilitate more acceptable and personal information exchange [1].

**Figure 1:**
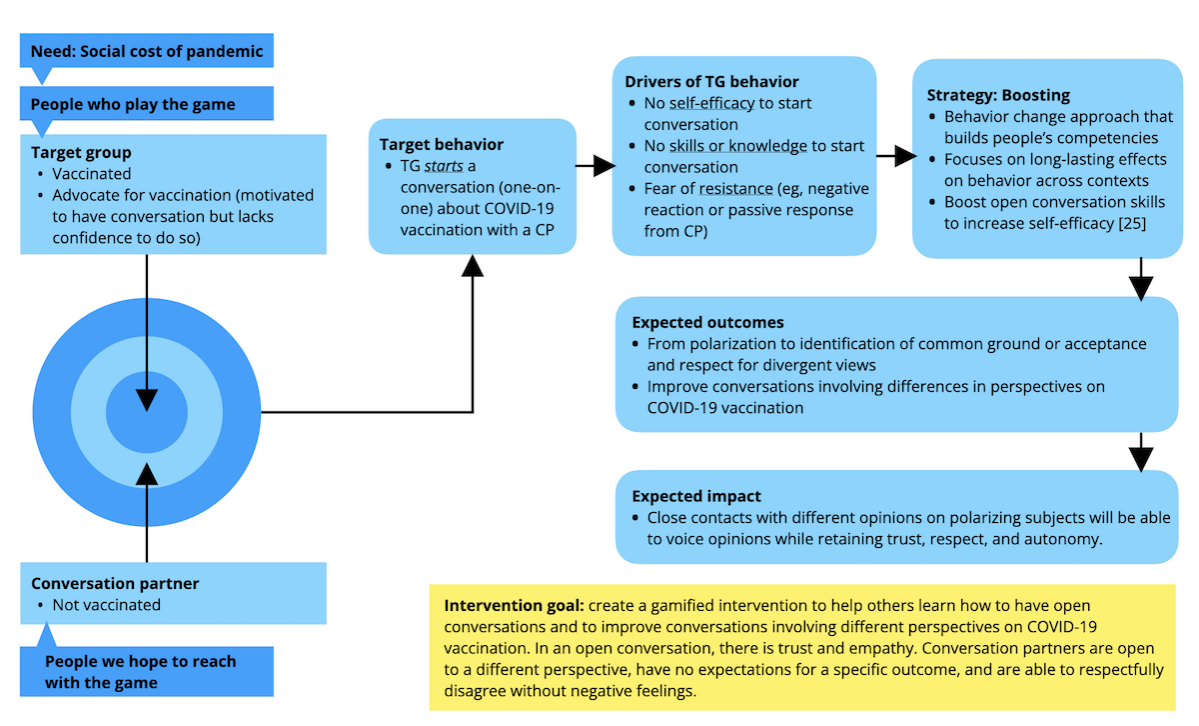
Theory of change for The Good Talk! intervention. CP: conversation partner; TG: target group.

Thus, the target group for the intervention is the close ties of vaccine-hesitant individuals who are vaccinated or are supportive of COVID-19 vaccination. For the purposes of this study, we refer to these individuals as *vaccine advocates*. As shown in the Theory of Change for the intervention (Figure 1), we aim to target vaccine advocates who are motivated to have a conversation about vaccination but lack the self-efficacy, skills, or knowledge to start a conversation or who fear resistance from their conversation partner. Motivation is an important driver of behavior change, and behavioral interventions are most effective when an individual is sufficiently motivated to learn new skills or competences [49,50]. In addition, targeting vaccine advocates increases the ecological validity and potential scope of the intervention, as they are likely to have more direct access and frequent encounters with vaccine hesitants and can reach broad and diverse audiences.

Accordingly, the goal of *The Good Talk!* intervention is to increase open conversations about COVID-19 vaccination by encouraging vaccine advocates to start a conversation with an individual they know or encounter who holds divergent or opposing views on vaccination or public health responses. We focus on *starting* an open conversation for several reasons. First, encouraging people to initiate a conversation is a first step to counter people’s motivations to avoid conversations about difficult topics (eg, owing to conflict concerns) [51,52]. Second, starting an open conversation is a concrete behavior as opposed to the more ill-defined concept of *having* a conversation and does not focus on the outcome of the conversation as an indicator of success. Although open conversations are important for building trust over the longer term, they may not lead to immediate changes in vaccination behavior or in clearly identifiable positive or negative outcomes following each conversation instance [53]. Thus, the aim of the intervention is to develop a participant’s competences, skills, and self-efficacy to approach rather than avoid conversations about COVID-19 vaccination. The intervention targets vaccine hesitancy indirectly by fostering more supportive interpersonal relationships between vaccine hesitants and vaccine advocates that encourage exposure to and consideration of divergent views. Ultimately, this should lead to continued exposure to divergent views between close ties and ongoing dialogue, which may eventually result in greater openness to the positions of others and remain open to evidence.

### Applying Behavioral Science to Improve COVID-19 Vaccine Communication

We applied the theoretical framework of boosting to guide the development of our intervention. Boosting is an evidence-based behavioral science approach that focuses on building people’s skills and competences rather than targeting a specific behavior [25,49]. The aim of boosting approaches is to foster people’s existing skills and competences or to develop new ones, thus helping individuals build their skills, knowledge, and self-efficacy to shift their motivations and translate their intentions or preferences into behavior [25,49]. A core feature of boosts is that they preserve people’s agency and enable them to exercise that agency to make their own choices [49]. Boosts are recommended for behavioral interventions where there is uncertainty or heterogeneity in people’s goals, when generalizability and longevity of a behavior is desired, and boosts are most effective when people are motivated to acquire new skills or competences [49,54]. Accordingly, a boosting approach is well suited to the design of an intervention that seeks to develop open conversation skills that are generalizable and flexible to addressing diverse causes of vaccine hesitancy, and for interventions that enable individuals to make their own choices on how to apply the skills to achieve their interpersonal relationship goals.

The focus of boosting approaches on fostering competences aligns with the objectives and key drivers of the intervention target behavior. Competence in interpersonal communication skills is associated with positive conversational outcomes, such as personal conversational satisfaction and more positive evaluations from conversation partners [55,56]. Demonstrating open conversation skills also leads to reduced defensiveness, greater awareness of attitude contradictions, decreased attitude extremity, and more open mindedness in conversation partners [57-59]. Furthermore, interventions designed to teach open conversation skills (eg, high-quality listening) or simple receptive conversation strategies have been found to signal positive collaborative intentions and receptiveness to alternative views while reducing negative conversational outcomes, such as defensiveness or threat reactions [57,59].

Developing open conversation skills can also build confidence and self-efficacy in conversations [60,61], and perceptions of self-efficacy are associated with conversational satisfaction [55]. Boosts foster self-efficacy by helping people learn and apply skills and knowledge through the development of competencies, rather than by providing information per se [25]. For instance, rather than teaching universal skills (eg, declarative knowledge), boosts use techniques such as teaching procedural rules, simple heuristics, or trainings (eg, learning how to harness implementation intentions) that can be generalized across different contexts or environments. The approach is conceptually similar to the inoculation approach used in misinformation detection interventions (eg, the *Bad News* game [23]), in which participants are exposed to the *techniques* that underlie misinformation and encouraged to generate their own antibodies rather than learn passively through being shown examples of manipulative strategies [62]. Learning through feedback on the effectiveness of skills can help build self-efficacy and confidence in understanding where and when to apply the skills to achieve one’s objectives [54].

Thus, our objective was to review the literature on open conversation skills to identify a set of simple strategies that could be integrated into a short behavioral intervention in the form of boosts. We aimed to design an intervention that would help individuals learn the effectiveness of these strategies on conversational outcomes through guidance and feedback, thus fostering participants’ self-efficacy in applying these strategies to initiate open conversations. In the following section, we will provide an overview of the evidence basis for the open conversation skills. The *Methods* section describes how we designed the intervention to provide participants with the opportunity to apply the strategies through an engaging and motivational serious game: *The Good Talk!*

### Open Conversation Skills

#### Overview

We conducted a narrative review of a broad range of literature on communication approaches to identify evidence-based open conversation skills that can be designed as boosts within the intervention. To align with the goals, we searched for communication literature that focused on developing or fostering supportive relationships, promoting self-efficacy and empowerment, or on psychological or therapeutic approaches developed to support behavior change. Specifically, we explored literature on the topics presented in Textbox 1.

**Communication literature reviewed to develop open conversation boosts.**

- Communication theories or frameworks to support behavior change (eg, motivational interviewing [7,63-65] and Self-Determination Theory [66-69])
- Core communication skills for fostering ongoing, supportive relationships (eg, building trust, empathy, and autonomy) [70-73]
- Literature on specific topics related to persuasive communication and listening skills [56-58,72] as well as strategies to increase receptiveness of divergent views and reduce psychological reactance (eg, conversational receptiveness) [59,74,75]
- Psychological strategies on how to handle negative reactions or outcomes (eg, mindfulness and intellectual humility) [76-78]
- Conversation guidelines or recommendations for health risk communications (eg, crisis communication) or on how to communicate about vaccinations [1,32,45,79-81]

From this literature, we identified 8 general open conversation skills that we categorized into 4 main communication objectives: to build trust and respect, be supportive, be receptive and demonstrate empathy, and practice acceptance. These objectives can be represented as a mnemonic for *The Good Talk!* acronym: gain trust, offer support, open up, and don’t be sad if it doesn’t work out. We provide a brief overview of each of the objectives and skills in the subsequent sections. More detailed descriptions of the open conversation skills and summaries of the supporting literature can be found in Table S1 in Multimedia Appendix 1 [7,56,58,59,63,65,66,69,72-76,79,82-87].

#### Gain Trust

The first objective focuses on building trust and rapport with the conversation partner to develop or maintain a supportive relationship or establish a partnership. The first of the 3 open conversation skills, *find mutual value*, aims to find points of agreement or shared value between conversation partners to create a sense of partnership, shared identity, or establish a common fate that one can connect or appeal to [69,79]. Finding points of agreement also signals receptiveness and positive collaborative intentions [59]. The second conversation skill, *respect autonomy*, aims to foster empowerment and self-efficacy in the conversation partner by treating them as responsible agents who have autonomy over their choices. Autonomy is considered a primary psychological need in Self-Determination Theory [66]. Further, emphasizing that one has and is responsible for their own choices reduces psychological reactance [69,75,82] and lowers feelings of defiance [83]. Fostering autonomy is a key recommendation in health risk and vaccine communication guides [53,79]. The third conversation skill, *don’t lecture or persuade (don’t be an expert)*, focuses on using language or strategies that avoid perceptions of authoritarian, coercive, or persuasive intent, which can lead to greater reactance to change communication [66,69,75,79,84]. For instance, using hedging (eg, use of “might” or “somewhat”) to soften factual claims has been found to signal collaborative intentions [59].

#### Offer Support

Offering support during a conversational exchange can facilitate relationship building and serves to foster self-efficacy in the conversation partner. The first conversation skill is to *ask for perspective* by showing interest in another’s viewpoint and expressing genuine appreciation for their concerns, feelings, or perspectives. Feeling understood and cared for by others is a primary psychological need according to Self-Determination Theory [66,69], and showing interest in another’s view leads to more positive evaluations of the conversation partner and greater receptiveness perceptions [56]. Affirmation of a conversation partner’s concerns is a component of motivational interviewing to promote self-efficacy and boost their confidence in taking action to make behavioral changes [7,63]. The second skill is to *affirm feelings/perspectives and acknowledge past efforts and successes* and is also a core component of motivational interviewing to support self-efficacy. It focuses on emphasizing the strengths, past successes, and efforts of the conversation partner and encouraging them to highlight their own strengths [7,63]. Applications of Self-Determination Theory to COVID-19 communication suggest that providing constructive feedback on how successful people have been in adhering to measures fosters a sense of competence and motivation to continue engaging in them [69].

#### Open Up

Two open conversation skills relate to the objective to open up. First, *asking open questions* aims to encourage individuals to use open (as opposed to closed) questions to gain insights and understanding and to allow their conversation partner to discuss their story. Open-ended questions are 1 of the 4 core communication skills in motivational interviewing to help establish a mutually trusting and respectful relationship with a conversation partner [7,63] and a strategy to accommodate people’s basic psychological need for relatedness in Self-Determination Theory [66,85]. Asking open questions is also an important active listening skill to enhance understanding and build relationships [73], and this communication skill is cited in numerous health risk communication guides [79,81]. Second, to *be resilient if the other* (conversation partner) *is right* promotes respect for divergent views (respectful disagreement) to foster open mindedness, shared identity, and more positive conversational exchanges [58,59,72]. At the same time, being willing to acknowledge and accept when a conversation partner is right or makes a valid argument demonstrates humility and openness to opposing views [76].

#### Don’t Be Sad If It Doesn’t Work Out

The final objective involves preparing the individual for a case in which their conversation will not necessarily lead to a positive outcome or observable behavior change [79]. The open conversation skill is to *accept negative outcomes* without judgment or negative evaluation of the situation or the conversation partner. Acceptance, nonjudgment, and nonreactivity are important for emotion regulation and for reducing strong negative emotions in mindfulness and acceptance and commitment therapy [70,78,86,88]. Accepting that the conversation may be one of several and leaving open the opportunity for future conversations can keep communication channels open and maintain ongoing relationships [79].

### Intervention Delivery: Serious Game

We sought to deliver the intervention through a medium that had a strong evidence basis for promoting behavior change, was realistic and had strong ecological validity in terms of interpersonal communications during the COVID-19 pandemic, would motivate people to engage with the intervention, and could reach broad and diverse audiences. Gamified behavioral interventions have been found to promote positive behavior change across a variety of health-related areas, including asthma control, medication adherence, and diet [89], and were found to be more effective than inactive controls or nongamified interventions in improving physical activity behavior [90]. Games have also been used as educational tools across a variety of fields [91,92], such as teaching students role-playing and perspective taking to improve open conversation skills [22], and have been found to be effective at inoculating people against disinformation [93,94], specifically COVID-19 disinformation [95].

Serious games are games that do not have “entertainment, enjoyment or fun as their primary purpose” [21]. The learning benefits of serious games align with the goals of boosting interventions, in which the aim is to boost competences and in which motivation is key to intervention efficacy [49]. Serious games increase knowledge, build skills, and increase self-efficacy through design features within the game play that help players to select relevant information and integrate and organize the information with prior knowledge [96]. For instance, a first-person perspective can engage players in the learning experience, narrative and choice instill a sense of autonomy, interactions with game characters and personal reflection provoke empathy and relationship or community building, and mistakes and feedback processes can foster persistence [92,96]. Furthermore, serious games not only enhance motivation to learn but also have a strong motivational appeal [92,96].

Serious web-based games have the potential to reach a broad and diverse audience, can be accessed worldwide on mobile and desktop devices, and can be shared with others via web-based media. Edutainment mobile games aimed at educating remote or low-resource communities on issues such as agriculture and farming practices, health, livelihoods, and financial literacy have been disseminated in over 20 countries, with COVID-19–focused games reaching >2.7 million people across 11 countries in 2020 [97]. Furthermore, as social distancing and safety measures during COVID-19 have limited close interpersonal contact and people have increasingly turned to web-based communication to stay connected [98], mimicking widely used web-based communication applications (eg, using a messenger-type app) [99] has strong ecological validity [100]. Another goal of using this format was to facilitate adaptation, translation, and implementation into existing public health channels that may be fighting misinformation via chatbots (eg, WHO’s Health Alert Services [101-103]), including in low-resource settings [104]. Therefore, we designed a serious game based on a messenger app to build self-efficacy and open conversation skills by mimicking real-world conversations between vaccine advocates and their close contacts. Further details on the specific design features of the serious game and how the open conversations skills were implemented are provided in the *Methods* section.

### Overview of Intervention Hypotheses

In the previous sections, we have detailed the theoretical development of *The Good Talk!* intervention. Our next step is to conduct a research study to evaluate the efficacy of *The Good Talk!* serious game to improve people’s skills and competences to have open conversations about COVID-19 with vaccine-hesitant individuals. In the following sections, we present a detailed research protocol describing the planned evaluation study to be conducted along with the implementation of the game. Once completed, the results of the evaluation study will be reported in a separate publication.

The efficacy of the intervention game will be evaluated in a preregistered randomized controlled trial. Specifically, we aim to answer the following research question: “How well does *The Good Talk!* game improve people’s open conversation skills, perceptions of self-efficacy, and behavioral intentions to have open conversations with vaccine-hesitant individuals who are close contacts?” We will compare a group of participants who play the intervention game with a control group that will play the widely known noneducational game *Tetris*. *Tetris* was selected as the game for the control condition as it is in the public domain, and most people know how to play it without the need for practice or instruction. *Tetris* involves a similar amount of cognitive effort to play as the intervention game, it contains no content related to the study, and it has been used as a control game to validate other digital game-based learning interventions [62,94,105]. *Tetris* is an inactive control group (receiving no educational material), which is an appropriate design for assessing the absolute efficacy of the intervention [106].

Our primary hypothesis is as follows:

- *Hypothesis 1: Relative to the control group, The Good Talk! game will result in a greater increase (boost) in participant’s skills to have an open conversation with a vaccine-hesitant individual*.

We also hypothesize the following:

- *Hypothesis 2: Relative to the control group, The Good Talk! game will result in a greater increase in individual’s feelings of self-efficacy to have an open conversation with a vaccine-hesitant individual*.
- *Hypothesis 3: Relative to the control group, The Good Talk! game will result in a greater increase in intentions to have an open conversation with a vaccine-hesitant individual*.

## Methods

### Participants and Recruitment

Participants will be recruited to play the game via video or push notifications promoting *The Good Talk!* game on social media platforms (eg, WHO chatbot channels) and advertised via other WHO informational campaigns. Recruitment will be open to all interested individuals worldwide but will be limited to English language speakers. Participants will self-subscribe to the study by accessing the WHO game information web page via the public link or when shared by members in their social network. On the game information page, participants will be asked if, in addition to playing the game, they would like to participate in the research element (a similar approach was used to recruit participants to research elements in other serious web-based games [93,95]). Participants who are not interested in participating in the study will be sent directly to play *The Good Talk!* game; otherwise, they will be asked to opt-in to the study after giving informed consent. Participation in the research element of the game will be restricted to individuals aged ≥18 years. Participation in the study is voluntary, and no remuneration will be offered to complete the study.

Previous studies evaluating the effect of similar serious games on the development of competences report small-to-medium effect sizes averaging around f=0.18 [95,100,105]. A power calculation with a small effect size (f=0.18), power of 0.90, and α=.05 for an analysis of covariance on posttest open conversation skills suggests a total sample size of n=327. We will aim to collect 450 responses (225 per condition) to account for potential technical issues or possible poor-quality data. Data collection will continue until the prespecified sample size for each condition is reached. Preregistration of this study can be found on the Open Science Framework [107].

### The Good Talk! Game

The intervention game was developed in collaboration with the game design company “Tiltstudio,” that has experience designing serious games including “Bad News,” “Go Viral,” and “Harmony Square,” targeting misinformation, COVID-19 misinformation, and political misinformation tactics, respectively. The game was designed to simulate messenger-type conversations between participants and close contacts in their social network. Messenger-type technologies are dyadic, informationally rich, synchronous, and mimic interpersonal communication and are therefore better suited for establishing a sense of connection and generating empathy [19]. At the same time, they represent a realistic communication platform familiar to many users worldwide [99], which increases the ecological validity of the intervention [100]. The game design implements instructional techniques found in a meta-analysis to be effective at improving learning and motivation in serious games, such as feedback (providing feedback or guidance on whether an answer is correct), reflection (participants are asked to reflect on their responses), level of realism (the game uses basic and cartoon-like representations), and narrative elements [96,108].

The game consists of 5 chapters in which the participants have conversations with characters representing their close contacts. In the first chapter, participants are introduced to the objective of the game, that is, to have as many people attend an event that they are organizing and to earn points for the good talk skills. The participant is asked to select an avatar to represent themselves and can choose between 1 of 4 social events they would like to organize (eg, birthday party, dinner, dance party, or housewarming).

**Figure 2:**
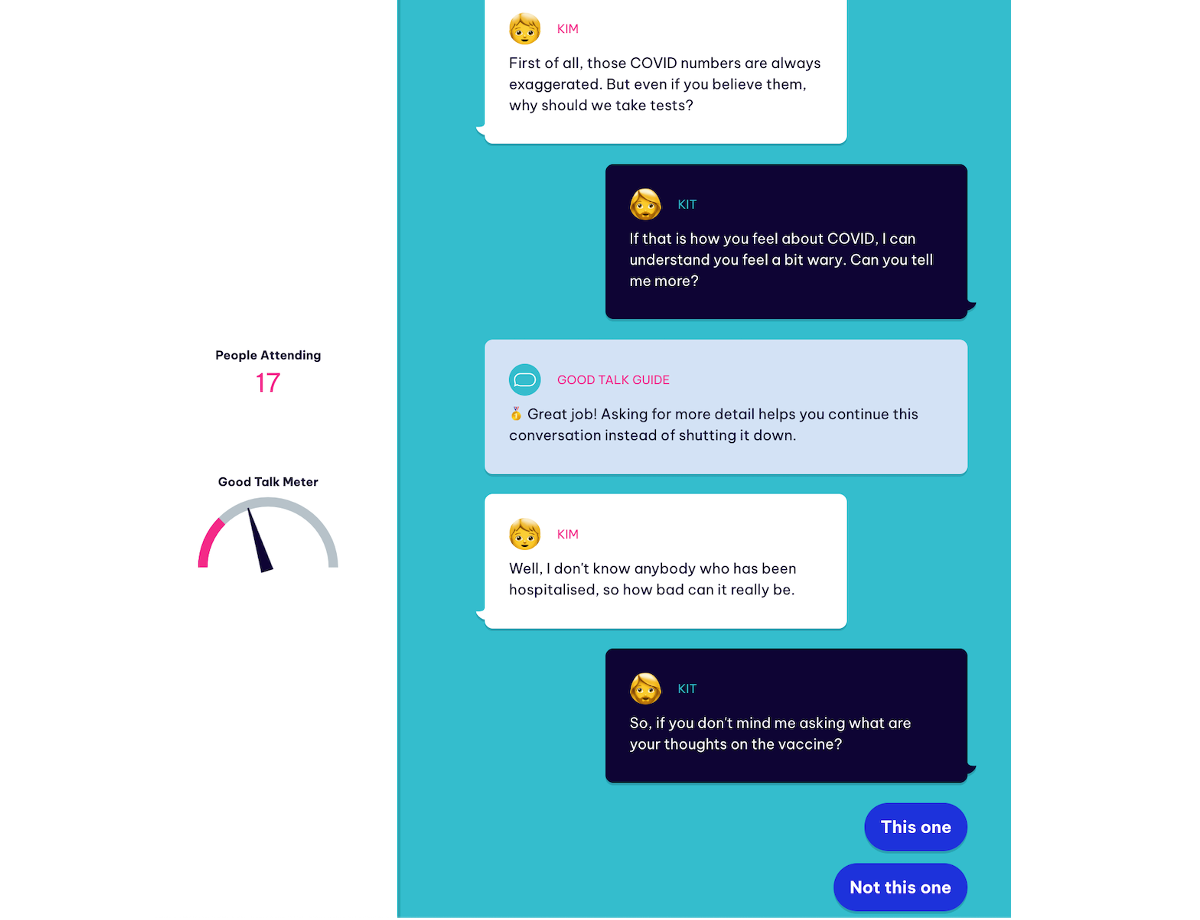
Example of The Good Talk! game interface on a web browser.

The remaining 4 chapters see the participants encounter characters within their close ties who are motivated by reactance (negative affective reaction when one feels personal freedoms are being threatened), skepticism (doubting knowledge or claims), and inertia (opposing change or action) when discussing COVID-19 measures for the social event. As the conversation develops, the player must select from ≥2 conversational responses that affect the participant’s pathway toward the game objective and the points they earn on a Good Talk Meter. The Good Talk Meter increases with response choices that represent good open conversation skills and decreases for those that represent bad open conversation skills. In each chapter, participants are guided by a *Good Talk Guide* who offers advice and reinforces or educates participants about the strategies based on their selections. A brief description of the objective of each chapter is provided in Table 1, and a screenshot of the game interface is shown in Figure 2.

**Table 1:** Brief overview of the goal and open conversation skills for each chapter.

Chapter	Goal
Introduction	The participant is introduced to the objective of the game and asked to select an avatar and an event they would like to plan. An overview of *The Good Talk!* skills is provided to participants along with the definition of what it means to have an open conversation.
Gain trust	The participant converses with someone who is worried about their kids not being vaccinated and whose kids are skeptical about the government’s use of COVID-19 antigen tests. The participant is encouraged to gain the trust of the conversation partner and, in turn, to encourage their partner to use open conversation skills to gain the trust of their children by expressing their concerns, finding mutual value, and supporting their kid’s autonomy.
Offer support	In this chapter, the conversation partner mistrusts the seriousness of the pandemic, is skeptical about the evidence, and does not see a personal need to get vaccinated. The participant is encouraged to ask for the perspective of their conversation partner rather than argue, with the goal to understand their views and to continue the conversation. By acknowledging their partner’s perspective and concerns, the participant is encouraged to find some common ground so that both feel comfortable attending the event.
Open up	The conversation partner is vaccinated but does not see a need to get the booster vaccination after reading some misinformation on the web and is reluctant to change their mind. The partner is overwhelmed by information and the impact of restrictions. By asking open questions, not being dismissive of their partner’s feelings or concerns, and accepting their points while being respectful of divergent views, the goal is to help the partner overcome their inertia.
Don’t be sad if it doesn’t work out	In the final chapter, the participant encounters a conversation partner who is against vaccination, who mistrusts the government, and who continues to share fake news and misinformation rather than engage in an open conversation. Rather than argue, the participant is guided to not react negatively and to accept that the conversation will be unlikely to lead to a positive outcome.

### Design

Participants who enter the game website and agree to participate in the research study will be randomized to the experimental or control group (between subjects) and complete both a pre- and postgame survey (within subjects). The experimental group will play *The Good Talk!* game, and the control group will play the noneducational game *Tetris*.

### Outcome Measures

#### Prior Experience With COVID-19 Vaccination

Participants will be asked whether they have had or are currently supportive of people getting the COVID-19 vaccination, whether they currently know someone who is hesitant toward getting the COVID-19 vaccination (and if so, their relationship with this individual), and whether they have previously tried to have a conversation about COVID-19 vaccination with a vaccine-hesitant individual.

#### Self-efficacy

Participants will rate how confident they are to be able to start a conversation about COVID-19 vaccination with a vaccine-hesitant individual on a 7-point scale ranging from “not at all confident” to “very confident.” The self-efficacy item will be asked in the pre- and poststudy surveys.

#### Behavioral Intention

Participants will be asked to indicate how likely it is that they will start an open conversation about COVID-19 vaccination with a vaccine-hesitant individual in the next 4 weeks on a 7-point scale ranging from “I will definitely not start a conversation” to “I will definitely start a conversation.” Consistent with recommendations for assessing intentions, the measure uses a likelihood scale and defines a timeframe for the behavior [109,110]. The behavioral intention item will be asked in the pre- and poststudy surveys.

#### Implementation Intention

Following the behavioral intention item in the poststudy survey, participants will be asked to plan their intention. Implementation intentions refer to intentions to enact a preplanned behavior and can mentally simulate the process of implementing a goal, aiding goal striving [110-112]. Participants will be asked to indicate which vaccine-hesitant individual they plan to have an open conversation about COVID-19 vaccination with (eg, a family member, friend, coworker, or someone else or indicate that they have no plan to have an open conversation with a vaccine-hesitant individual), when they plan to have the conversation (eg, in the next ___ hours/days/months), and to specify what their starting sentence will be.

#### Open Conversation Skills

Participants will be provided with conversation snippets presented as messenger app messages and asked to rate each statement according to how good or bad they think the response is when having an open conversation on a 5-point scale ranging from “very bad” to “very good.” Participants will rate 8 conversation snippets in the prestudy survey and a different set of 8 conversation snippets in the poststudy survey. Using parallel versions of pre- and postintervention tests in the evaluation of serious games reduces practice effects [106,113]. In each survey, there will be 1 conversation snippet for each of the 8 open conversation skills (Table 2). For each open conversation skill, one item will represent a bad response example and the other will represent a good response example. Good and bad response examples for a single open conversation skill will not appear in the same survey. The open conversation skill snippets were reviewed by experts in behavioral science interventions.

**Table 2:** Examples of good and bad open conversation skills included in the pre- and poststudy measure.

Objective and skill		Good response	Bad response
**Build trust**			
	Find mutual value	I am worried about the safety of the vaccine for my family. What if it made them sick?^a^ Response: I’m concerned about my family’s health too and also feel responsible if something bad was to happen to them.	Hey, so I’m unvaccinated but I’m still gonna go over to my friend’s house who is really strict about people being vaccinated. Response: Hmn, that’s not something I would do. I would respect other’s decisions.
	Respect autonomy	I can’t believe the government is suggesting that everyone should get vaccinated. This is my health, my decision. Response: The choice is yours and only you can make it. What would help you make your decision?	These COVID regulations are ridiculous, next thing you know I’ll have to wear a hazmat suit to go shopping.^a^ Response: You should respect other people’s health and follow the regulations.
	Don’t lecture or persuade (don’t be an expert)	How can they even be sure the vaccine is safe if they made it so fast? It was rushed, who knows what steps they missed!^a^ Response: I think it might have something to do with reducing all the red tape that helped speed things up but I’m not sure. Let’s look into it together!	I am concerned about the side-effects of the vaccine. I heard that they can get pretty bad. Response: Have you looked at the data? I have, and the side-effects are minor. You should look into what can happen if you don’t get the vaccine.
**Offer support**			
	Ask for perspective	My friend has a party but she says I need to be vaccinated to go, not sure how I feel about it? Response: What makes you unsure?	I don’t believe getting a booster vaccine is really necessary.^a^ Response: It’s interesting how you justify putting other people at risk.
	Affirm feelings or perspective; acknowledge past efforts or successes	I’m unsure about whether to take the vaccine, it seems dangerous.^a^ Response: It is good that you are critical and don’t rush into things when you are unsure. Remember when you had to switch to that new medication and felt better when you looked into it more?	Our neighbours were strict with the social distancing thing and one of them got it anyway. And they are both vaccinated! So I just don’t see the point. Response: That’s not how to think about it. Just because they got it doesn’t mean it’s not worth you making any effort.
**Open up**			
	Ask open questions	For now, I don’t want to get the vaccine. Just because everyone else has decided to get it, doesn’t mean that I have to as well. Response: If you were to consider getting the vaccine, what would change your mind?	How would a mask even help against a virus?^a^ Response: Did you look at the evidence?
	Be resilient if the other is right	First we were told not to bother with masks, and then we were told to wear masks everywhere! These backflips don’t help.^a^ Response: That’s true, the changing recommendations about wearing masks did not help. It goes to show how uncertain things were at the beginning.	I’m waiting, that’s all. It takes time to make sure that the vaccine is safe for everyone. Response: Yeah I’ve heard that point before but let’s focus on why it is important that all healthy people should get vaccinated.
**Don’t be sad if it doesn’t work out**			
	Accept negative outcomes (the conversation may not work out)	It’s clear that you and I are not on the same page about this. I have my reasons, you have yours. Response: That’s OK, thanks for helping me understand your reasons. It seems like it doesn’t work out, so let’s leave it there and maybe we can speak again next week?	Look I just don’t think we are ever going to agree on this.^a^ Response: You don’t appreciate my views! But give me one more try and I bet I can change your mind on this one.

^a^Items included in the prestudy survey.

#### Demographic Questions

Participants will be asked to provide their age (in years) and indicate their gender, current level of English fluency, country of residence, and highest level of education obtained.

### Procedure

Participants will first answer the questions regarding their support for COVID-19 vaccination and their past experience speaking with vaccine-hesitant individuals. Participants will then complete a pretest survey containing the 8 open communication skills items and the self-efficacy and behavioral intention items. The experimental group will then play *The Good Talk!* game, which will take approximately 10 minutes to complete. The control group will play *Tetris* for a similar amount of time.

**Figure 3:**
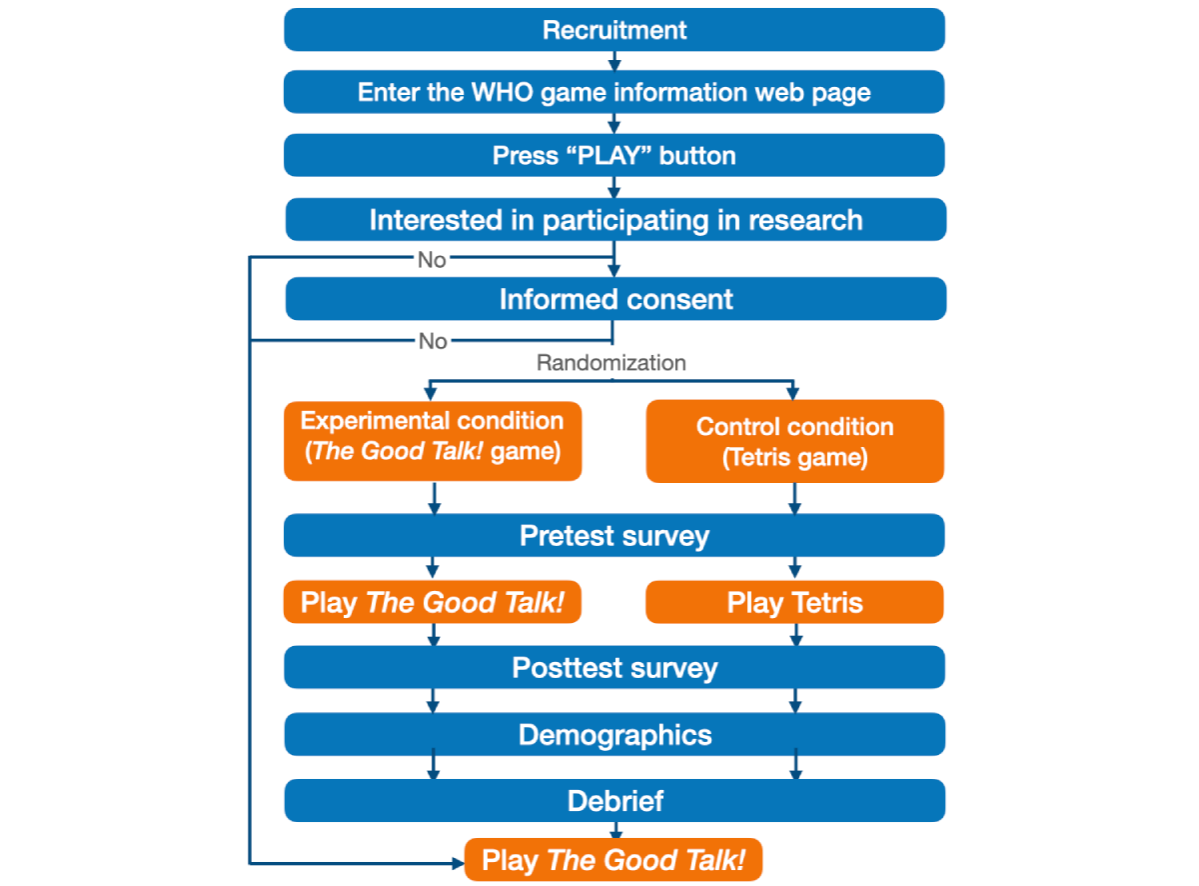
Flow diagram representing the study design and procedure. WHO: World Health Organization.

Following the game, participants will respond to a posttest survey containing the open communication skills, self-efficacy, and behavioral intention questions. Participants will also complete the implementation intention questions and a short demographic survey. At completion of the study, participants will be thanked for their time and invited to share the study with their friends. Participants in the control group will be given the option to play *The Good Talk!*. Figure 3 presents the flowchart of the study design and procedure.

### Ethics Approval

Ethics approval for the study has been submitted and is awaiting approval from the WHO Ethics Review Committee (application ID: CERC.0177).

## Results

Recruitment and data collection are estimated to commence in early 2023. The efficacy of the study will be analyzed using an analysis of covariance with posttest open conversation skills as the dependent variable, controlling for pretest scores. Partially completed surveys will be included in statistical analysis whenever possible. As a sensitivity check, analyses will be repeated by excluding participants who complete the study less than 2 SDs below the median completion time as a data quality check.

The primary outcome is open conversation skills. The secondary outcomes are self-efficacy and behavioral intention items. Exploratory analyses will examine the effect of the game on implementation intentions as well as potential covariates or subgroup differences based on sociodemographic information or previous experiences with COVID-19 vaccination conversations.

## Discussion

### Expected Outcomes

We hypothesize that *The Good Talk!* game will lead to a greater increase in open conversation skills, self-efficacy, and intentions to initiate conversations about vaccination between vaccine advocates and their vaccine-hesitant close ties relative to the control game. We hope that the game will help people to talk to one another so that supportive relationships can be maintained, and over time, opportunities to share with consideration of divergent views may help combat some of the negative social costs of the pandemic. Furthermore, we hope that this study will highlight how serious games can be a promising forum for delivering evidence-based health behavior interventions to the public.

### Strengths and Limitations

*The Good Talk!* game represents an evidence-based behavioral science application based on the theoretical framework of boosting that aims to boost people’s skills and competences rather than target a specific cause of vaccine hesitancy. As such, this approach has the potential to build transferrable skills that can be applied across a variety of situations. Delivering the intervention via a worldwide, publicly available serious game means that it has the potential to reach a broad and diverse audience while using an ecologically valid medium that mimics real-world web-based communications.

Nevertheless, there are potential limitations to the insights that can be drawn from this study. First, as participants will access the study via a public link that is associated with the promotion of the game, it is not possible to blind participants completely from their assigned experimental condition. Although participants will be blinded to their assigned conditions at the initial pretest survey stage, it is not possible to avoid dropouts or potential self-selection to their assigned condition. To limit this effect, all participants will be told that both games are very important for the research and that everyone will get the opportunity to play *The Good Talk!* game. We will also collect data on the number of dropouts and partially complete and completed surveys per condition to evaluate their impact on study completion. Second, the game may attract a diverse set of participants from various backgrounds worldwide, including different levels of English proficiency, experience with COVID-19 vaccination, and social and political contexts. However, as the intention for the game intervention is to reach a broad and diverse audience and be applicable to individuals from a wide range of cultural, economic, and educational backgrounds, the evaluation will also contribute data on the effectiveness of the intervention in a real-world context.

### Future Directions

We envisage that the positive results of the study can lead to concrete practical applications, for instance, as a valuable resource for health care professionals whose patients request advice on how to have vaccination conversations with those close to them. Furthermore, health care systems can refer to *The Good Talk!* game to boost the competencies of their patients to have open conversations about other difficult topics with their close contacts. More broadly, we hope that this study can motivate health care policies to consider serious games such as ours as an additional and efficacious method for promoting behavior change in public health communications.

### Conclusions

*The Good Talk!* intervention represents an application of boosting theory to the field of behavior change for public health purposes. By describing the methods through which the intervention was designed, we hope to enable others to replicate and innovate our findings. We hope our approach will encourage more health ministers to engage in their mission to reach their citizens directly with digital health solutions and to consider such interventions as an important tool in infodemic management.

## Appendix

Multimedia Appendix 1 Overview of open conversation boosts and supporting literature.

## Abbreviations

WHO: World Health Organization
