# Supplementary material for: The Good Talk! A Serious Game to Boost People’s Competence to Have Open Conversations About COVID-19: Protocol for a Randomized Controlled Trial
Source: JMIR Res Protoc. 2023 Mar 8;12:e40753. doi: 10.2196/40753 (PMC9997707; doi:10.2196/40753)
Supplement: Multimedia Appendix 1 [file resprot_v12i1e40753_app1.docx]

## Supplementary Material

Table S1. Overview of Open Conversation Boosts and Supporting Literature

| **Objective** | **Skill** | **Description** | **Selected Supporting Literature** | **Theoretical Background** |
| --- | --- | --- | --- | --- |
| Gain trust | Find mutual value | Find points of agreement or shared value; emphasise and facilitate shared identity and common fate | - Emphasise and facilitate shared identity and common fate; appeal to the goals, and values of the individual [69] - Create a culture of partnership [65] - Find points of agreement [59]; elicit and connect with people’s values [79] | Motivational Interviewing; Self Determination Theory;  Conversational receptiveness; Health risk communication guides |
|  | Respect autonomy | Treat people as responsible agents; provide sense of choice; foster empowerment and self-efficacy | - Autonomy support (feeling of being the origin of one’s own behaviour) is a primary psychological need [66] - Treat people as responsible agents and provide a sense of choice, where possible [65,69,79] - Offering alternative behaviour option reduces psychological reactance as it reduces threat to freedom [75,82] - Autonomy-supportive messages about social distancing more effective at lowering feelings of defiance [83] | Motivational Interviewing; Self Determination Theory; Conversational receptiveness; Psychological reactance; Vaccination communication; Health risk communication guides |
|  | Don’t lecture or persuade (don’t be an expert) | Foster a partnership that is supportive rather than persuasive; hedge to soften claims; do not be coercive, authoritarian or persuasive | - Foster a partnership that is supportive rather than persuasive [7] - Avoid control, do not use coercive, authoritarian or guilt-inducing language [66,69] - Do not use forceful or explicit messages, or demonstrate clear intent to persuade [75] - High controlling language (should, ought, must) produces greater reactance to attitude change communications [84] - Hedge (e.g., use of “might” or “somewhat”) to soften factual claims) [59] | Motivational Interviewing, Self Determination Theory; Conversational receptiveness; Psychological reactance |
| Offer support | Ask for perspective | Show expressed interest in another’s viewpoint to foster receptiveness; acknowledge people’s perspectives, feelings and potential conflicts | - Affirm partner’s concerns or limitations - express genuine appreciation and positive regard to promote self-efficacy and boost confidence about taking action [7,63] - Showing expressed interest in another's viewpoint leads to more positive evaluations of the conversation partner and greater receptiveness [56] - Involvement/relatedness (feeling understood and cared for by others) is a primary psychological need [66,69] | Motivational Interviewing; Self Determination Theory; Health risk communication guides |
|  | Affirm feelings/  perspective, acknowledge past efforts, successes | Affirm perspectives and feelings; emphasise strengths, past successes, and efforts to take small steps | - Emphasise strengths, past successes and efforts to take small steps; encourage the individual to highlight their strengths [7,63,65] - Support self-efficacy: Discuss previous situations in which the individual was successful [7] - Provide constructive, clear, and relevant feedback on how successful people have been in adherence to measures [69] - Acknowledge barriers and obstacles people have by engaging in relevant behaviours [69] | Motivational Interviewing; Self Determination Theory |
| Open up | Ask open questions | Help the partner discuss their story; pay attention and gather knowledge; use as an alternative to counter-arguing to gain perspective | - Asking open questions to let one’s partner discuss their story and fears is a core communication and active listening skill [7,73,79,84] - Attunement (paying attention and gathering knowledge about a person) is part of people’s involvement/ relatedness psychological needs [66,85] - Question-asking can offer similar affective benefits to counter-arguing without the potential negative effects of conflict escalation [74] | Motivational Interviewing; Self Determination Theory; Conversational receptiveness; Response to disagreement; Active listening |
|  | Be resilient if the other is right | Offer a respectful disagreement about issues, recognize and appreciate knowledge and intellectual strength of others | - Respect for divergent values (respectful disagreement) leads to more positive conversational exchanges [59,] - Individuals perceive a greater shared identity with close ties when they express respect for divergent values (e.g., political views) [72] - Perceived responsiveness of a communicator fosters open-mindedness and promotes awareness of opposing attitudes [58] - Willingness to accept one’s own ignorance/lack of knowledge associated with increased number of respectful attributions made in a disagreement and openness to opposing views [76] | Intellectual humility; High-quality listening skills; Accommodating communication (respect for divergent values) |
| Don’t be sad if it doesn’t work out | Accept negative outcomes (the conversation may not work out) | Accept without judgement, be non-judgemental or non-evaluative about present-moment experience | - Accept that there may be several conversations and that one conversation is unlikely to change a person’s mind [79] - Component of mindfulness is non-judgement and non-reactivity for emotion regulation [87] - Encourage acceptance when a person is faced with unwanted experiences such as aversive affect or cognitions [86] | Mindfulness; Acceptance and Commitment Therapy; Health risk communication guides |
